# Immunopathogenesis of brain abscess

**DOI:** 10.1186/1742-2094-1-16

**Published:** 2004-08-17

**Authors:** Tammy Kielian

**Affiliations:** 1 Department of Neurobiology and Developmental Sciences, University of Arkansas for Medical Sciences, Little Rock, Arkansas 72205, USA

**Keywords:** brain abscess, *S. aureus*, microglia, astrocytes, neuroinflammation

## Abstract

Brain abscess represents a significant medical problem despite recent advances made in detection and therapy. Due to the emergence of multi-drug resistant strains and the ubiquitous nature of bacteria, the occurrence of brain abscess is likely to persist. Our laboratory has developed a mouse experimental brain abscess model allowing for the identification of key mediators in the CNS anti-bacterial immune response through the use of cytokine and chemokine knockout mice. Studies of primary microglia and astrocytes from neonatal mice have revealed that *S. aureus*, one of the main etiologic agents of brain abscess in humans, is a potent stimulus for proinflammatory mediator production. Recent evidence from our laboratory indicates that Toll-like receptor 2 plays a pivotal role in the recognition of *S. aureus *and its cell wall product peptidoglycan by glia, although other receptors also participate in the recognition event. This review will summarize the consequences of *S. aureus *on CNS glial activation and the resultant neuroinflammatory response in the experimental brain abscess model.

## Pathogenesis of brain abscess

Brain abscesses develop in response to a parenchymal infection with pyogenic bacteria, beginning as a localized area of cerebritis and evolving into a suppurative lesion surrounded by a well-vascularized fibrotic capsule. The leading etiologic agents of brain abscess are the *streptococcal *strains and *S. aureus*, although a myriad of other organisms have also been reported [1,2]. Brain abscess represents a significant medical problem, accounting for one in every 10,000 hospital admissions in the United States, and remains a serious situation despite recent advances made in detection and therapy [2]. In addition, the emergence of multi-drug resistant strains of bacteria has become a confounding factor. Following infection, the potential sequelae of brain abscess include the replacement of the abscessed area with a fibrotic scar, loss of brain tissue by surgical excision, or abscess rupture and death. Indeed, if not detected early, an abscess has the potential to rupture into the ventricular space, a serious complication with an 80% mortality rate [1]. The most common sources of brain abscess are direct or indirect cranial infection arising from the paranasal sinuses, middle ear, and teeth. Other routes include seeding of the brain from distant sites of infection in the body (i.e. endocarditis) or penetrating trauma to the head. Following brain abscess resolution patients may experience long-term complications including seizures, loss of mental acuity, and focal neurological defects that are lesion site-dependent.

**Figure 1:**
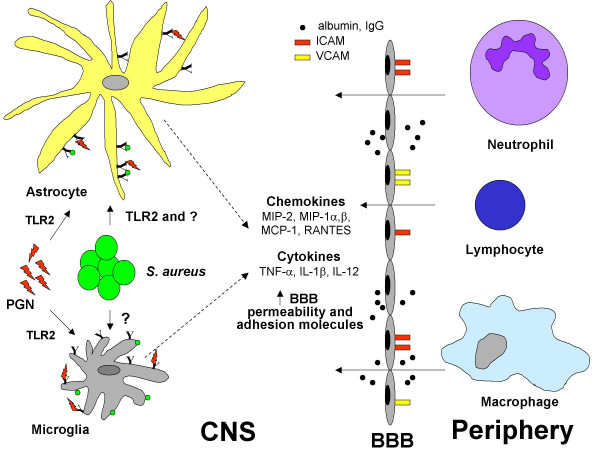
Immunopathogenesis of brain abscess. Pyogenic bacteria such as *S. aureus *induce a localized suppurative lesion typified by direct damage to CNS parenchyma and subsequent tissue necrosis. Bacterial recognition by Toll-like receptor 2 (TLR2; **Y**) leads to the activation of resident astrocytes and the elaboration of numerous proinflammatory cytokines and chemokines. Microglia produce a similar array of proinflammatory mediators following bacterial stimulation; however, the receptor(s) responsible for *S. aureus *recognition and subsequent cell activation remain to be identified. Both microglia and astrocytes utilize TLR2 to recognize peptidoglycan (PGN) from the bacterial cell wall. Proinflammatory cytokine release leads to blood-brain barrier (BBB) compromise and the entry of macromolecules such as albumin and IgG into the CNS parenchyma. In addition, cytokines induce the expression of adhesion molecules (ICAM, intercellular adhesion molecule; VCAM, vascular cell adhesion molecule) which facilitate the extravasation of peripheral immune cells such as neutrophils, macrophages, and T cells into the evolving abscess. Newly recruited peripheral immune cells can be activated by both bacteria and cytokines released by activated glia, effectively perpetuating the anti-bacterial immune response that is thought to contribute, in part, to disease pathogenesis.

At the histological level, brain abscess is typified by a sequential series of pathological changes that have been elucidated using the experimental rodent models described in detail below [3-7]. Staging of brain abscess in humans has been based on findings obtained during CT or MRI scans. The early stage or early cerebritis occurs from days 1–3 and is typified by neutrophil accumulation, tissue necrosis, and edema. Microglial and astrocyte activation is also evident at this stage and persists throughout abscess development. The intermediate, or late cerebritis stage, occurs from days 4–9 and is associated with a predominant macrophage and lymphocyte infiltrate. The final or capsule stage occurs from days 10 onward and is associated with the formation of a well-vascularized abscess wall, in effect sequestering the lesion and protecting the surrounding normal brain parenchyma from additional damage. In addition to limiting the extent of infection, the immune response that is an essential part of abscess formation also destroys surrounding normal brain tissue. This is supported by findings in experimental models where lesion sites are greatly exaggerated compared to the localized nature of bacterial growth, reminiscent of an over-active immune response [5,8,9]. This phenomenon is also observed in human brain abscess, where lesions can encompass a large portion of brain tissue, often spreading well beyond the initial focus of infection. Therefore, controlling the intensity and/or duration of the anti-bacterial immune response in the brain may allow for effective elimination of bacteria while minimizing damage to surrounding brain tissue. The mechanisms elucidated to date in the immunopathogenesis of brain abscess are depicted in Figure 1.

## *S. aureus*-induced experimental brain abscess model

Although case reports of brain abscess in humans are relatively numerous, studies describing the nature of the ensuing CNS and peripheral immune responses are rare. Therefore, our laboratory has developed a mouse experimental brain abscess model to elucidate the importance of host immune factors in disease pathogenesis [5,7-9]. Our mouse model was modified based on a previously published model in the rat [3] and utilizes *S. aureus*, one of the main etiologic agents of brain abscess in humans. The mouse brain abscess model accurately reflects the course of disease progression in humans, providing an excellent model system to study immunological pathways influencing abscess pathogenesis and the effects of therapeutic agents on disease outcome. We have successfully utilized this model to characterize inflammatory mediators induced in the brain immediately following *S. aureus *exposure [5] as well as identification of bacterial virulence factors critical for pathogenesis *in vivo *[8]. For example, we have demonstrated that *S. aureus *leads to the immediate and sustained expression of numerous proinflammatory cytokines and chemokines in the brain including tumor necrosis factor-alpha (TNF-α), interleukin-6 (IL-6), IL-1α,β, macrophage inflammatory protein-2 (MIP-2/CXCL2), monocyte chemoattractant protein-1 (MCP-1/CCL2), MIP-1α/CCL3, MIP-1β/CCL4, and regulated upon activation T cell expressed and secreted (RANTES/CCL5) [5,7-9].

As mentioned earlier, lesion sites in both our experimental model and in human brain abscess are greatly exaggerated compared to the localized nature of bacterial growth, reminiscent of an over-active immune response. To account for the enlarged region of affected tissue involvement associated with brain abscesses compared to the relatively focal nature of the initial insult, we have proposed that proinflammatory mediator production following *S. aureus *infection persists, effectively augmenting damage to surrounding normal brain parenchyma [10]. Specifically, the continued release of proinflammatory mediators by activated glia and infiltrating peripheral immune cells may act through a positive feedback loop to potentiate the subsequent recruitment and activation of newly recruited inflammatory cells and glia. This would effectively perpetuate the anti-bacterial inflammatory response via a vicious pathological circle culminating in extensive collateral damage to normal brain tissue. Recent studies support persistent immune activation associated with experimental brain abscesses with elevated levels of IL-1β, TNF-α, and MIP-2 detected from 14 to 21 days following *S. aureus *exposure [9]. Concomitant with prolonged proinflammatory mediator expression, *S. aureus *infection was found to induce a chronic disruption of the blood-brain barrier, which correlated with the continued presence of peripheral immune cell infiltrates and glial activation [9]. Collectively, these findings suggest that intervention with anti-inflammatory compounds subsequent to sufficient bacterial neutralization may be an effective strategy to minimize damage to surrounding brain parenchyma during the course of brain abscess development, leading to improvements in cognition and neurological outcomes.

Besides the potential detrimental roles cytokines may exert on surrounding normal brain parenchyma during the later stages of brain abscess, numerous proinflammatory cytokines such as IL-1β, TNF-α, and IL-6 may have beneficial effects on the establishment of host anti-bacterial immune responses. These cytokines exert numerous functions within CNS tissues including modulation of blood-brain barrier integrity, induction of adhesion molecule expression on cerebral microvascular endothelial cells, and subsequent activation of resident glia and infiltrating peripheral immune cells [11-17]. We recently examined the relative importance of IL-1, TNF-α and IL-6 in experimental brain abscess using cytokine knockout (KO) mice [7]. The IL-1 KO animals used for these studies were deficient in both IL-1α and IL-1β; therefore, potential caveats arising from redundancy in the activities of these two proteins were avoided. Despite the fact that these cytokines share many overlapping functional activities, IL-1 and TNF-α appear to play an important role in dictating the ensuing anti-bacterial response in brain abscess. This was evident by the finding that bacterial burdens were significantly higher in both IL-1 and TNF-α KOs compared to wild type mice which correlated with enhanced mortality rates in both KO strains [7]. In contrast, IL-6 was not found to be a major contributor to the host anti-bacterial immune response. These studies established important roles for IL-1 and TNF-α during the acute phase of experimental brain abscess development, indicating that these cytokines individually dictate essential functions for the establishment of an effective anti-bacterial response in the CNS parenchyma.

Neutrophils are potent bactericidal effector cells and represent the major peripheral cell infiltrate associated with developing brain abscesses [5,9]. Neutrophils exert their bactericidal activity through the production of reactive oxygen and nitrogen intermediates and hydrolytic enzymes that directly destroy bacteria. In addition, neutrophils serve as a source of proinflammatory cytokines, such as TNF-α that serve to amplify the host anti-bacterial immune response [18,19]. However, the continuous release of these products by newly recruited and activated neutrophils can also contribute to tissue damage. Therefore, depending on the context of inflammation, neutrophils can have either beneficial or detrimental effects on the course of infectious diseases. We have recently revealed the functional importance of neutrophils in brain abscess development using antibody-mediated neutrophil depletion and CXCR2 KO mice where neutrophils lack the high-affinity receptor for the neutrophil chemoattractants MIP-2/CXCL2 and KC/CXCL2 [5]. Interestingly, in spite of elevated levels of the CXCR2 ligands MIP-2 and KC, neutrophil extravasation was impaired in CXCR2 KO mice, with cells remaining sequestered within small vessels in developing brain abscesses. Impaired neutrophil influx into evolving brain abscesses in both CXCR2 KO and neutrophil-depleted mice led to exacerbated disease typified by elevated bacterial burdens compared to wild type animals [5]. These studies demonstrate that CXCR2 ligands are the major chemotactic signals required for neutrophil influx into brain abscesses and that their activity cannot be substituted by alternative chemotactic factors such as complement split products (i.e. C3a, C5a), prostaglandins, leukotrienes, or other chemokines. Similar to our findings, the importance of neutrophils in *S. aureus*-induced acute cerebritis was demonstrated by Lo et al. where transient neutrophil depletion resulted in enhanced pathology [20]. In addition to MIP-2 and KC, numerous other chemokines are also detected within evolving brain abscesses including MIP-1α, MIP-1β, MCP-1, and TCA-3/CCL1 [5,8]. The potential roles these chemokines play in the pathogenesis of brain abscess development remain to be defined. However, they could be envisioned to influence the accumulation of monocytes and lymphocytes into the brain and possibly the establishment of adaptive immune responses. Indeed, we and others have demonstrated the influx [21](Kielian, unpublished observations) and generation of *S. aureus*-specific lymphocytes [9] in experimental brain abscess.

Staphylococci produce a wide array of virulence determinants that play a role in disease pathogenesis [22,23]. These can be broadly subdivided into surface and extracellular secreted proteins. Surface proteins include structural components of the bacterial cell wall such as lipoteichoic acid and peptidoglycan. Secreted proteins are generally expressed during the exponential phase of bacterial growth and include such proteins as α-toxin, lipase, and enterotoxin. We recently reported that virulence factor production by *S. aureus *is essential for the establishment of brain abscess in the experimental mouse model [8]. Specifically, a requirement for ongoing bacterial replication and/or virulence factor production was supported by the finding that heat-inactivated bacteria were not sufficient to induce proinflammatory cytokine/chemokine expression or abscess formation in the brain. Using a series of *S. aureus *mutants with various defects in virulence factor expression, we identified α-toxin as a critical virulence factor determinant in the experimental brain abscess model. Replication of a *S. aureus *α-toxin mutant was significantly attenuated in the brain, which correlated with a reduction in proinflammatory mediator expression and the failure to establish a well-defined abscess [8]. We proposed that in wild type bacteria, α-toxin, which leads to pore formation in mammalian cell membranes and subsequent osmotic lysis, serves as an effective mechanism to eliminate CNS resident immunocompetent cells (i.e. microglia and astrocytes) as well as professional phagocytes that infiltrate brain abscesses and exert potent anti-bacterial activity (i.e. neutrophils and macrophages). This would effectively impair the efficacy of the ensuing anti-bacterial immune response, allowing bacterial burdens to expand unchecked during the acute phase of disease. In contrast, in the absence of α-toxin secretion, resident glia and infiltrating leukocytes would be capable of rapidly neutralizing bacteria, effectively facilitating the resolution of infection in a timely manner and thus preventing the establishment of a well-formed abscess. However, it is likely that additional virulence factors participate in *S. aureus *infection in the brain since the α-toxin mutant was not completely avirulent. Potential candidates include V8 protease, staphylococcal enterotoxin B, and protein A, the latter of which has been shown to bind to TNF receptor I in the host [24].

Recently, the *S. aureus*-induced experimental brain abscess model has been utilized by Stenzel et al. to demonstrate an important role for astrocytes in dictating the extent of brain abscess pathology [21]. Using glial fibrillary acidic protein (GFAP) KO mice, this group showed that brain abscess pathogenesis was exacerbated in KO animals where lesions were larger and typified by ill-defined borders, severe brain edema, and enhanced levels of vasculitis compared to wild type mice. In addition, GFAP KO mice exhibited a diffuse leukocyte infiltrate that extended into the uninfected contralateral hemisphere. Exacerbation of brain abscess severity in GFAP KO mice was attributed to the absence of a bordering function by astrocytes to contain the infection since strong GFAP immunoreactivity was observed along the abscess margins in wild type animals. It is intriguing that the absence of GFAP influences brain abscess evolution in such a dramatic manner, as astrocytes are still present and functional in these mice. It is possible that GFAP expression in activated astrocytes induces structural changes that influence the local cytoarchitecture leading to bacterial dissemination in brain abscess.

Collectively, the studies to date performed in the mouse experimental brain abscess model have begun to elucidate critical mediators in the pathogenesis of disease and host cytokines that play a pivotal role in the generation of the CNS anti-bacterial immune response. However, there are numerous issues that remain to be resolved regarding the role of inflammatory mediators in the evolution of brain abscess. For example, the potential importance of other proinflammatory cytokines and chemokines detected in brain abscess remain to be defined. In addition, factor(s) that participate in the initiation of the anti-bacterial adaptive immune response remain to be elucidated. Evidence to support the establishment of an adaptive immune response is provided by our recent findings that *S. aureus*-specific lymphocytes are formed during the later stages of experimental brain abscess development [9]. It is not known whether the immune response generated during a previous brain abscess episode is capable of providing protection against a second CNS challenge. Another question relates to the potential dual role of various proinflammatory mediators during the course of brain abscess pathogenesis. As mentioned above, a dual role for IL-1 and TNF-α has been suggested by our findings that these cytokines are critical for establishing an effective host anti-bacterial immune response during the acute stage of brain abscess development. However, IL-1 and TNF-α expression persists within brain abscesses for at least 14 to 21 days following infection, suggesting an over-active immune response that is not down-regulated in a timely manner. We are currently using knockout mice to investigate the potential dual role these cytokines may exert during the evolution of brain abscess. Addressing these issues may facilitate the design of effective therapeutic regimens for brain abscess that would be capable of pathogen elimination without the accompanying destruction of surrounding brain parenchyma that normally occurs in disease.

## Responses of microglia to the brain abscess pathogen *S. aureus*

Relevant to our experimental brain abscess model, recent studies from our laboratory have established that both intact *S. aureus *and its cell wall product peptidoglycan (PGN) serve as potent stimuli for proinflammatory mediator production in primary microglia [5,10,25]. Specifically, exposure to both stimuli led to a dose- and time-dependent induction of the proinflammatory cytokines IL-1β, TNF-α, IL-12 p40, and several chemokines including MIP-2, MCP-1, MIP-1α, and MIP-1β. The importance of microglia in the early host response to infection in brain abscess is suggested by the fact that proinflammatory mediator production is detected within 1 to 3 hours following the initial *S. aureus *infection, well before the significant accumulation of peripheral immune cell infiltrates [4]. Another study has also demonstrated that *S. aureus *induces IL-1β expression in neonatal rat microglia [26].

Microglia represent one of the main antigen presenting cells in the CNS [11,27]. To achieve efficient activation of antigen-specific T cells, microglia must express sufficient levels of major histocompatability complex (MHC) class II (signal I) and co-stimulatory molecules such as CD40, CD80, and CD86 (signal II). Recognition of signal I without the concomitant engagement of signal II results in T cell non-responsiveness or anergy. Our group found that both heat-inactivated *S. aureus *and PGN are capable of inducing microglial MHC class II [10,25], CD40, CD80, and CD86 receptor expression, similar to what has been described for microglia in response to the gram-negative bacterial product lipopolysaccharide (LPS) and interferon-γ (IFN-γ) [27-31]. The ability of *S. aureus *to augment the expression of receptors that are important for antigen presentation suggests that the ability of microglia to present bacterial peptides to antigen-specific T cells may be greatly enhanced following an initial exposure to *S. aureus*. The effects of *S. aureus *and PGN on microglial CD40, CD80, CD86, and MHC class II expression may either be a direct consequence of bacterial stimulation or indirect via the autocrine action of cytokines produced by activated microglia.

Microglial activation is a hallmark of brain abscess [4,5,9]. They respond robustly to both *S. aureus *and PGN with significant proinflammatory mediator expression, and many of these same mediators are persistently elevated in brain abscess. Drawing on this relationship, we have proposed that chronic microglial activation may contribute, in part, to the excessive tissue damage characteristic of brain abscess. Therefore, attenuating chronic microglial activation subsequent to effective bacterial elimination in the brain may result in attenuation of the structural and functional damage associated with brain abscess. We have recently examined the efficacy of the cyclopentenone prostaglandin 15d-PGJ_2 _to modulate microglial responses to *S. aureus *[10]. 15d-PGJ_2 _was found to be a selective and potent inhibitor of *S. aureus*-dependent microglial activation through its ability to significantly attenuate the expression of numerous proinflammatory cytokines and chemokines of the CC family including IL-1β, TNF-α, IL-12 p40, MCP-1, and MIP-1β. In addition, 15d-PGJ_2 _also selectively inhibited the *S. aureus*-dependent increase in microglial TLR2, CD14, MHC class II, and CD40 expression whereas it had no effect on the co-stimulatory molecules CD80 and CD86. The ability of 15d-PGJ_2 _to modulate the expression of these receptors may serve as a means to regulate microglial and T cell activation during gram-positive bacterial infections in the CNS. Preventing microglial activation by 15d-PGJ_2 _or related compounds may help to resolve inflammation earlier, resulting in reductions in brain abscess size and associated damage to surrounding normal brain parenchyma.

## Receptors utilized by microglia for bacterial recognition

As detailed above, our laboratory has established that microglia are capable of recognizing *S. aureus *and respond with robust production of numerous proinflammatory mediators. However, to date, the receptor repertoire responsible for bacterial recognition remains to be defined. In macrophages, numerous receptors have been implicated in bacterial phagocytosis and subsequent activation leading to proinflammatory mediator release including Toll-like receptors (TLR), scavenger receptors, and mannose receptors. The fact that microglia and macrophages share many functional and phenotypical characteristics supports the contention that these receptors may play an important role in microglial responses to bacteria.

Toll-like receptors are a family of surface receptors expressed on cells of the innate immune system that allow for the recognition of conserved structural motifs on a wide array of pathogens (referred to as pathogen-associated molecular patterns) [32,33]. To date, eleven TLR have been identified, with TLR2 playing a pivotal role in recognizing structural components of various gram-positive bacteria, fungi, and protozoa [34]. Several groups have reported TLR2 expression in microglia, with receptor expression augmented following inflammatory activation [25,35-38]. Relevant to brain abscess, we have demonstrated that both *S. aureus *and PGN lead to significant increases in TLR2 mRNA and protein expression, which may enhance microglial sensitivity to bacteria during the course of experimental brain abscess development [25]. Recent studies from our laboratory using primary microglia from TLR2 KO mice have revealed that TLR2 plays a pivotal role in recognition of PGN but not intact *S. aureus *(Kielian, manuscript in preparation). These findings indicate that an alternative receptor(s) is involved in mediating responses to intact bacteria. Candidates include the mannose receptor and members of the scavenger receptor family.

Scavenger receptors encompass a broad range of molecules involved in receptor-mediated phagocytosis of select polyanionic acids such as lipoteichoic acid of *S. aureus *[39]. Although adult microglia do not express scavenger receptors in the normal CNS, their expression is induced following inflammation or injury [40]. In the context of brain abscess, a potential tripartite role for microglial scavenger receptors can be envisioned that would include regulating cell adhesion and retention within the inflammatory milieu, facilitating bacterial phagocytosis, and promoting the removal of apoptotic cell debris associated with the evolving abscess [41]. Preliminary data suggest that *S. aureus *and PGN differentially modulate the expression of several distinct scavenger receptors that may influence the nature and extent of phagocytosis (Kielian, unpublished observations). Scavenger receptors have been implicated in β-amyloid phagocytosis by microglia in the context of Alzheimer's disease, in part, by the finding that microglia associated with senile plaques express a high degree of scavenger receptor immunoreactivity [42,43]. In addition, scavenger receptors have been implicated in β-amyloid uptake by microglia [44-47]. The functional importance of scavenger receptors in *S. aureus *phagocytosis by microglia remains to be established.

Microglia have been shown to express functional mannose receptors that are responsible for the binding and phagocytosis of mannosylated and fucosylated ligands of bacteria [48,49]. Interestingly, proinflammatory cytokines such as IFN-γ and LPS have been shown to downregulate mannose receptor expression on microglia [48,49]. Using microarray analysis, we also recently demonstrated that mannose receptor levels were significantly attenuated in microglia following *S. aureus *exposure, suggesting that the regulation of mannose receptor expression is conserved among diverse stimuli [25]. Following the subsequent internalization of molecules via the mannose receptor by antigen presenting cells, an immune response can be generated in either a MHC class I, class II, or CD1-restricted manner [50-52]. In addition, some studies have indicated a functional coupling of the mannose receptor to microbiocidal activities, strongly suggesting a cytotoxic activity linked to mannose receptor-ligand interactions [53]. The functional importance of mannose receptors in the initial recognition and phagocytic events in microglia following *S. aureus *exposure remain to be defined. In addition to the receptors described above, there are additional candidates that may serve as receptors for *S. aureus *phagocytosis in microglia including complement receptor 3 (also known as CD11b/CD18) and CD14, the latter of which we have shown to be expressed on microglia and significantly upregulated following activation with either *S. aureus *or PGN [10,25].

## Responses of astrocytes to the brain abscess pathogen *S. aureus*

Astrocytes play a pivotal role in the type and extent of CNS inflammatory responses. These cells likely play an important role in the initial recruitment and activation of peripheral immune cells into the CNS during neuroinflammation through the production of several cytokines and chemokines, such as IL-1, IL-6, IL-10, TNF-α, IFN-α/β, granulocyte-macrophage colony-stimulating factor (GM-CSF), macrophage-CSF (M-CSF), granulocyte-CSF (G-CSF), transforming growth factor-beta (TGF-β), RANTES, MCP-1, and IFN-γ-inducible protein-10 (IP-10/CXCL10) [12,54].

Various studies have documented the ability of LPS to induce nitric oxide (NO), cytokine, and chemokine production in astrocytes [55,56]. In contrast, the characterization of products produced by astrocytes following exposure to gram-positive bacteria had remained largely undefined until recently. Studies from our group have revealed that primary astrocytes are capable of recognizing both intact *S. aureus *and PGN and that they respond with vigorous proinflammatory cytokine and chemokine production [57]. Among the factors produced by *S. aureus*-activated astrocytes are NO, TNF-α, IL-1β, MIP-2, MCP-1, MIP-1α, and MIP-1β. These proinflammatory chemokines may serve as signals for neutrophil (MIP-2), monocyte and lymphocyte (MCP-1, MIP-1β) recruitment *in vivo*, whereas IL-1β and TNF-α likely alter blood-brain barrier permeability and induce the expression of critical adhesion molecules on CNS vascular endothelium required for immune cell extravasation into brain abscesses.

## Receptors utilized by astrocytes for bacterial recognition

Astrocytes have recently been shown to express TLR2 [38,58], and although these cells are capable of responding to the well-characterized TLR2 ligand PGN [58], the functional significance of this receptor was not directly demonstrated until recently. Using primary astrocytes from TLR2 KO and wild type mice, our laboratory was the first to report that TLR2 plays a pivotal role in the recognition of *S. aureus *and PGN and in subsequent cytokine and chemokine expression by astrocytes [57]. Interestingly, the production of these cytokines and chemokines was only partially attenuated in TLR2 KO astrocytes, suggesting that alternative receptors are also involved in bacterial recognition. There are numerous candidates for alternative receptors in astrocytes for gram-positive pathogens like *S. aureus*. For example, TLR2 has been shown to form functional heterodimers with TLR1 and/or TLR6 [59,60], thereby increasing its range of antigen detection. It has recently been suggested that CD14 serves as a co-receptor for TLR2 [61] and enhances the recognition efficiency of many TLR2-specific ligands including PGN and lipoteichoic acid [62-64]. Recently, several studies have reported data that support the involvement of additional, as of yet uncharacterized pattern recognition receptors in bacterial recognition [61,65]. Alternatively, activation through mannose and scavenger receptors that play an important role in the phagocytic uptake of bacteria and have been reported to be expressed by astrocytes [66-68] may be responsible for the residual proinflammatory mediator expression in TLR2 KO astrocytes. However, to date, the functional importance of these alternative receptors in mediating astrocyte activation in response to *S. aureus *and PGN is currently not known.

Although astrocytes have been shown to possess phagocytic activity in response to β-amyloid [69], apoptotic cells [70], and yeast [71,72], the phagocytic potential of astrocytes is still a subject of controversy. Data from our laboratory indicates that primary astrocytes are capable of phagocytosing *S. aureus *[57]. An active phagocytic process is supported by the finding that astrocytes rapidly internalize heat-killed *S. aureus*, indicating that bacterial uptake occurs via a phagocytic pathway and is not simply the result of productive infection by live organisms. Interestingly, TLR2 is not a major receptor for bacterial phagocytosis in astrocytes since both TLR2 KO and wild type astrocytes were equally capable of phagocytosing intact *S. aureus *organisms *in vitro *[57]. The receptor(s) responsible for mediating bacterial uptake in astrocytes are not known but could include the mannose and/or scavenger receptors described above. Studies to identify receptors responsible for *S. aureus *phagocytosis by astrocytes and the optimal conditions required for bacterial uptake are currently ongoing in our laboratory. Issues such as whether bacterial internalization is serum-dependent or requires other bacterial binding proteins must also be addressed.

## Conclusions and perspectives

The incidence of brain abscess is expected to persist in the human population due to the ubiquitous nature of bacteria coupled with the recent emergence of antibiotic-resistant bacterial strains. Therefore, understanding the roles of both host anti-bacterial immune responses along with bacterial virulence factors may lead to the establishment of novel therapeutic treatments for brain abscess. The mouse *S. aureus *experimental brain abscess model provides an excellent tool for deciphering the importance of various mediators in disease pathogenesis. Especially appealing is the ability to examine the role of specific factors using transgenic and knockout mice because, in our experience, all of the mouse strains examined with this model have qualitatively similar inflammatory profiles following bacterial challenge. In addition, the consequences of *S. aureus *infection do not appear to be influenced by gender, as the responses of female and male mice are similar- another advantage when performing studies with knockout or transgenic mice where animal numbers are often limiting.

The responses of microglia and astrocytes to *S. aureus *have been elucidated in terms of proinflammatory mediator expression and in general, have been found to be qualitatively similar to those observed following LPS exposure. Although studies with primary microglia and astrocytes from TLR2 KO mice reveal an important role for this receptor in mediating *S. aureus*-dependent activation, it is clear that additional receptors are also involved in glial responses to this bacterium. This functional redundancy is not surprising because these pathogens have the potential for devastating consequences in a tissue that has limited regenerative capacity such as the CNS.

The implications of glial cell activation in the context of brain abscess are likely several-fold. First, parenchymal microglia and astrocytes may be involved in the initial recruitment of professional bactericidal phagocytes into the CNS through their elaboration of chemokines and proinflammatory cytokines. Second, microglia exhibit *S. aureus *bactericidal activity *in vitro*, suggesting that they may also participate in the initial containment of bacterial replication in the CNS. However, their bactericidal activity *in vitro *is not comparable to that of neutrophils or macrophages, suggesting that this activity may not be a major effector mechanism for microglia during acute infection. Third, activated microglia have the potential to influence the type and extent of anti-bacterial adaptive immune responses through their upregulation of MHC class II and co-stimulatory molecule expression. Finally, if glial activation persists in the context of ongoing inflammation, the continued release of proinflammatory mediators could damage surrounding normal brain parenchyma. Indeed, inappropriate glial activation has been implicated in several CNS diseases including multiple sclerosis and its animal model experimental autoimmune encephalomyelitis as well as Alzheimer's disease. The continued use of transgenic and knockout mice for *in vivo *studies will facilitate our understanding of immune mechanisms contributing to brain abscess pathogenesis.

## List of abbreviations

BBB blood-brain barrier

CCL CC chemokine ligand

CD cluster of differentiation

CSF cerebral spinal fluid

CXCL CXC chemokine ligand

CXCR CXC chemokine receptor

GFAP glial fibrillary acidic protein

GM-CSF granulocyte-macrophage colony-stimulating factor

IFN interferon

IL interleukin

IP-10 interferon-inducible protein-10

KO knockout

LPS lipopolysaccharide

M-CSF macrophage colony-stimulating factor

MCP monocyte chemoattractant protein

MHC major histocompatability complex

MIP macrophage inflammatory protein

NO nitric oxide

PGN peptidoglycan

RANTES regulated upon activation T cell expressed and secreted

TGF transforming growth factor

TNF tumor necrosis factor

## Competing interests

None declared.
